# Hemp cultivation opportunities for marginal lands development

**DOI:** 10.1371/journal.pone.0299981

**Published:** 2024-03-21

**Authors:** Elisa Scalabrin, Marta Radaelli, Gabriele Capodaglio, Manuela Pierobon, Silvia Del Vecchio, Gabriella Buffa

**Affiliations:** 1 National Research Council, Polar Science Institute, Venice-Mestre, Italy; 2 Department of Environmental Sciences, Informatics and Statistics, University of Venice, Ca’ Foscari, Venice-Mestre, Italy; 3 Department of Biological, Geological and Environmental Sciences, Alma Mater Studiorum University of Bologna, Bologna, Italy; University of Brescia: Universita degli Studi di Brescia, ITALY

## Abstract

Agricultural diversification and high-quality products deriving from sustainable crops such as hemp can represent a solution to revitalize marginal areas and reverse land abandonment. This study aimed at comparing four different hemp cultivars (Carmagnola Selezionata, “CS”; Futura 75, “FUT”; Felina 32, “FEL”; Secuieni Jubileu, “JUB”) to provide information to select the best suited cultivar for cultivation in mountain marginal areas and for specific end-use applications. Hemp cultivars were cultivated in a single experimental field to compare their ecological and agronomic behavior (duration of life cycle phases, plant size and biomass allocation, and plant resource-use strategies). Through metabolomic analysis of both vegetative and reproductive parts of the plants we tested the presence of substances of nutraceutical interest and traced seed nutritional profile. The four cultivars had different ecological and agronomic behavior, and nutritional profile. We found several compounds with potential pharmaceutical and nutraceutical values in all parts of the plant (leaves, inflorescences, and stems). JUB resulted the most suitable for seed production while CS showed the highest content of bioactive compounds in flowers and leaves. FUT, showed the best suitability for multi-purpose cultivation, while FEL seemed to be not appropriate for the cultivation in mountain area. The multi-disciplinary approach we adopted was effective in distinguish across hemp cultivars and provided information to farmers for the selection of the best hemp cultivar to select. Hemp had a high potential for cultivation in marginal lands, demonstrating to be an economic resource due to its multi-purpose use and to the possibility to generate high-added values products. Our results could serve as a stimulus for the reintroduction of this culture in the study area and in other similar environments.

## Introduction

Marginal lands, characterized by biophysical and socio-economic constraints that make conventional cultivation economically impractical, encompass underproductive, vulnerable, and abandoned areas [1]. The abandonment of marginal lands in western Europe started in the 1950s/1960s and persists today. Projections indicate that by 2030, approximately 11% of the EU’s agricultural land will face a high or very high potential risk of abandonment, while the conversion of abandoned land back to agriculture is expected to be negligible at 0.11% [2]. Land marginalization and abandonment have wide implications, including a general loss of local biodiversity [3], as well as social and environmental problems in areas with few economic alternatives [4]. Although the utilization of marginal lands for conventional agriculture is often economically inconvenient, marginal lands are frequently essential for the conservation of such ecosystem services as biodiversity, soil protection, climate regulations, especially in the context of a warming climate [5].

To prevent marginal land abandonment, we need a proactive approach able to provide alternative perspectives and uses [6]. Marginal lands, unsuitable for conventional crops, have great potential for transformation [1], and recultivating these lands provides opportunities for diversification and high-quality productions [4,7]. Marginal and abandoned areas can thus be used to test unconventional development models and creative ideas for innovative forms of entrepreneurship, contributing to the balanced development of rural areas by enhancing their unique territorial characteristics [8]. This approach aligns with the European Green Deal’s goal of fostering a competitive economy that exploits available resources for sustainable development.

Hemp (*Cannabis sativa* L.) is an annual crop that combines rapid growth, environmental sustainability and high versatility [9,10]. It requires limited technical inputs, no pesticides or herbicides [11] since it naturally and very effectively suppresses weeds; hemp cultivation also contributes to soil improvement [9,12], including heavy metal contaminated soil [13]. According to Adesina et al. [14], hemp can be grown in a great variety of agro-ecological conditions and is able to grow quickly, especially in the first weeks after emergence, making it an excellent candidate for carbon sequestration [15]. Moreover, hemp can be regarded as a multi-purpose crop since its biomass can be used in a whole range of applications: fiber for the production of textiles and ropes, shives for bio-building construction materials, seeds for high-quality oil or other food products, leaves for pharmaceutical purposes [16,17]. In recent years, there has been also an increased focus on the nutritional qualities of seeds as far as fatty acid [18] and protein content [19] are concerned, as well as on the potential pharmacological effects of hemp inflorescences [20].

In Italy, hemp was widely cultivated until the 1950s [21], also in mountain and hilly areas, mainly for the production of technical textiles [22]. However, the problematic presence of psychoactive substances produced by some cultivars, coupled with the progressive diffusion of synthetic fibers, the increasing cost of labor, the difficulties in mechanizing cultivation and the greater economic advantages of other crops led to the gradual decline of hemp cultivation in the whole Europe during the 20th century. In recent years, the growing recognition of hemp’s diverse applications and its positive environmental impact has increased its appeal for modern agriculture [20]. The regulation of hemp cultivation in Italy through law n. 242 of 2/12/16, including its use in food and nutraceutical sectors, supported and promoted hemp production chain in Italy [23]. The legislation permits only certified cultivars with THC content < 0.2%, according to National and European Regulations [24,25]. While regulations establish the maximum THC level in food products [26], the legal commercialization of hemp flowers remains unaddressed. The striking properties of hemp cultivars represent a potential solution for reversing marginal land abandonment, offering a model for multi-output systems and for the creation of all-round supply chains [10,27]. The development of integrated micro-chains, based on industrial hemp use, has certainly a potential positive impact on the territory, involving different production sectors (agriculture, trade, and industry) and applicative outcomes (e.g., naturalistic engineering and phytoremediation).

However, since hemp is a genetically diverse and variable crop that produces a range of raw products of diverse nature [14], it is crucial to understand basic cultivar biology and ecology and test the suitability of hemp cultivation in marginal lands, highlight its economic and environmental potentiality, and demonstrate the possibility of obtaining high-added value products. In light of this, the aim of this study was to compare four different hemp cultivars in order to provide information for the selection of the best suited cultivar for cultivation in mountain marginal areas and for specific end-use applications. To this aim, hemp was cultivated in a single experimental field, representative of marginal areas in the Italian pre-alpine region. To evaluate the best end-use application of each cultivar we compared their ecological and agronomic behavior, namely plant phenology (i.e., duration of life cycle phases), plant structure (e.g., plant size and biomass allocation), and plant resource-use strategies. To investigate the hemp potential for revaluing food product quality from a nutraceutical point of view we performed a metabolomic analysis of the different parts of the plants (both vegetative and reproductive) to test the presence of bioactive substances of nutraceutical interest and trace seed nutritional profile of each cultivar.

## Materials and methods

### Study area

The research was conducted in the province of Belluno, a prevalently mountain area in the eastern Alps of Italy. The area presents a continental climate, with annual average rainfall of 1400 mm and average monthly temperatures ranging between -5°C in January and +23°C in July [3]. The study area was located in Ponte nelle Alpi (latitude: 46°07’47.6" North; longitude: 12°10’59.4" East; altitude: 414 m a.s.l.), an area highly representative of the process of depopulation and land marginalization and abandonment. Until 2–3 decades ago, the area was in full production, but recent years have seen remarkable changes in land use that led to a decreasing number of farms and a decreased surface of cultivated land mostly due to socio-economic and biophysical factors, such as lack of crop competitiveness, small size farmlands, bad accessibility to land. From 1982 to 2010, the reduction of the total agricultural area was around 50%, the utilized agricultural area decreased of 32% on average, and the number of agricultural farms reduced of 84% [28,29].

### Field experiment

The field experiment was carried out in 2017, by sowing in open fields four cultivars of *Cannabis sativa*: Carmagnola Selezionata (CS), Futura 75 (FUT), Felina 32 (FEL), Secuieni Jubileu (JUB). Table 1 summarizes their characteristics like origin, sexual type, and main usage purposes. The cultivars have been selected for their low THC content, in compliance with the legal limits. Additionally, they were selected to cover a spectrum of single-, dual-, or multi-purpose use, aligning with those most commonly reported in the literature (e.g., Secuieni Jubileu for grain vs Felina 32 for grain, fiber, and CBD; Table 1; [30,31]).

**Table 1:** Cultivars.

Cultivar name	Acronym	Origin	Sexual type	Sowing date	Sowing density (kg/ha)	Usage
Carmagnola Selezionata	CS	Italy	Dioecious	19/05/2017	50	Fiber/CBD^a^
Futura 75	FUT	France	Monoecious	19/05/2017	50	Grain/CBD^b^
Felina 32	FEL	France	Monoecious	19/05/2017	50	Grain/Fiber/CBD^b^
Secuieni Jubileu	JUB	Romania	Monoecious	19/05/2017	50	Grain^b^

Cultivar name, acronym, origin, sexual type, sowing date and density (kg/ha), main usage of the four cultivars considered in this study. Grain (seed); Fiber (hurd); CBD (inflorescence). a: [30]; b: [31].

**Fig 1:**
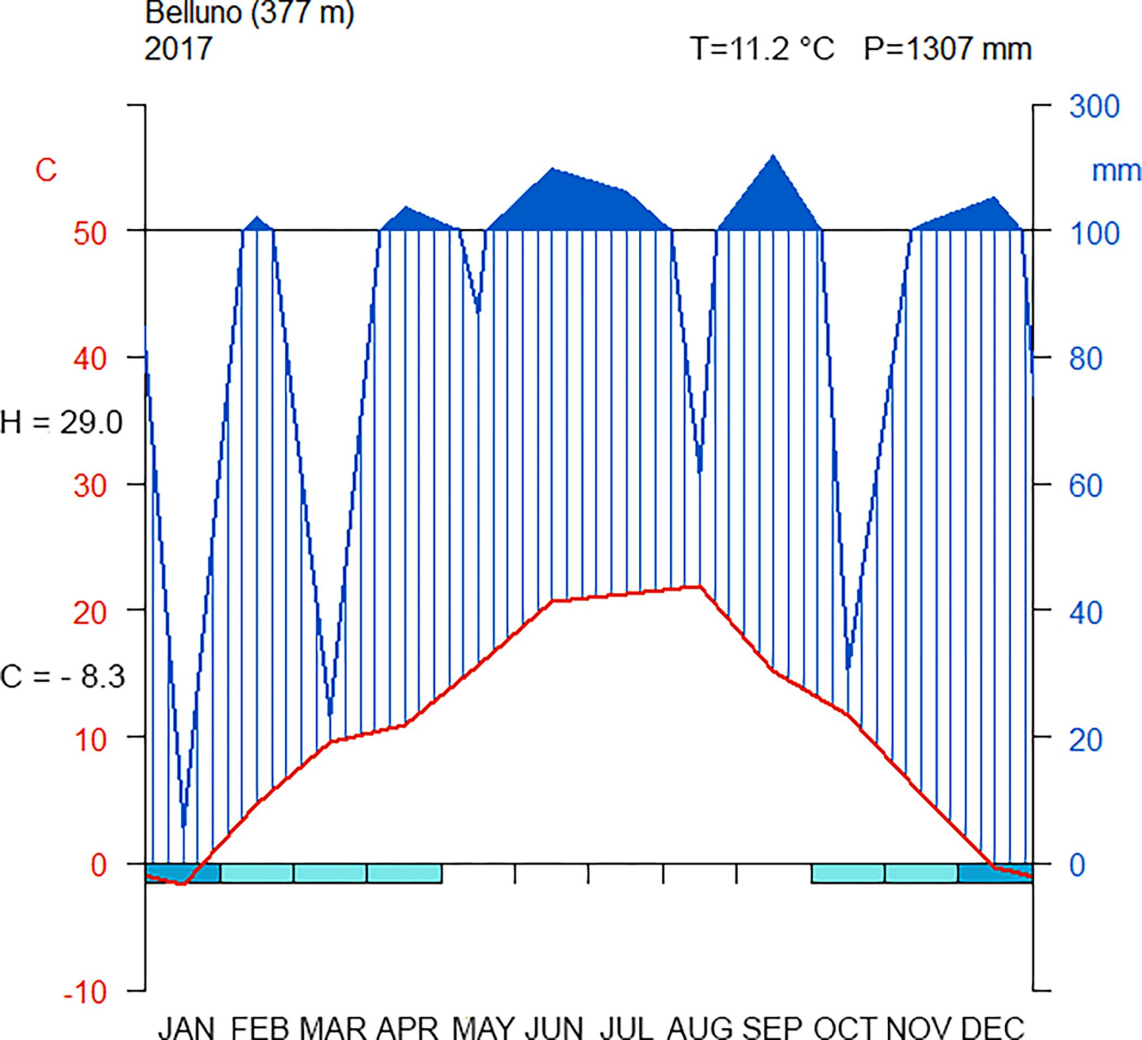
Termopluviometric diagram. Termopluviometric diagram for climatic station of Belluno (Latitude = 5117458, Longitude = 1750560, Gauss-Boaga ovest). The blue line is the total precipitation for each month in 2017, while the red line is the mean temperature. The blue bars on the month axis indicates periods of possible frost. The black values on the temperature axis (y, left) are the averages of the hottest (“H”) and coldest (“C”) month. The values on the right upper corner are the average annual temperature (“T”) and the total annual precipitation (“P”).

The four cultivars were grown in a single gently sloped (1–4%) experimental field, to assure the same environmental conditions, without any treatment or fertilization. Each cultivar was sowed in plots of 5 m^2^ and the sowing was performed on the same day and with the same density. Weather conditions during the field experiment are summarized in Fig 1. On average, soils of the study area have medium profile differentiation, moderate depth (50–100 cm), high carbonate (10–25%) and skeleton (15–35%) contents, and medium texture [32]. All methods and sample collection fully align with the relevant legislation. No permits were required to approve access to the field site, as one of the authors owns the field.

### Life cycle, biomass allocation, resource-use strategy

After emergence, in each plot, we recorded plant density. Plant density is related to the ability to suppress weeds [4,33] and to other plant structural characteristics related to hemp products; e.g., despite contrasting weeds efficiently, a high plant density can reduce plant height, stem diameter, and plant total biomass. Plant density was calculated by counting all the plants in 1 m^2^ at 60 days after sowing. Other parameters descriptive of the plant morphology, as the internode lengths, the number of leaves, and the seed size, mass, shape and number are provided in the Supporting information (S1 Table).

#### Life cycle phases

The duration of life cycle phases influences the plant structure, since it can affect stem size [34], seed yield [35], and the biomass allocation (i.e., vegetative vs. reproductive allocation). To analyze life cycle phases, each plot was monitored every 15 days to detect (i) the emergence delay, as the days elapsed between sowing and seedling emergence, as the appearance of the first pair of leaves. After emergence, in each plot we randomly chose 20 individuals to measure (ii) the duration of vegetative growth, corresponding to the period from emergence to flowering; (iii) flowering duration, as the number of days between the beginning and the end of flowering; (iv) seeds formation, from the beginning of seed formation to seed maturation. At the maximum development of the plants, i.e., when seeds were mature, we measured the plant height from the soil surface to the base of the inflorescence (i.e., inflorescence excluded), and stem, measured at the 4^th^ internode.

#### Biomass allocation

In order to determine the biomass allocation, 10 (up to 13, when possible) randomly chosen individuals were harvested at seed maturity (when at least 50% of the seeds were hard). The samples were separated into root, stem, inflorescences, and seeds. The dry weight of root, stem, and inflorescences was measured after drying at 75°C for 70 h. Seeds (i.e., achenes) were picked off the plant, counted (to obtain the number of seeds/plant) and stored under controlled conditions (18.5°C and 40% relative humidity). The seed mass was quantified as the air-dry weight of 100 seeds [36].

To analyze the relative allocation of biomass within the plant parts, regardless of the plant size [37], we calculated the ratio among the dry weight of roots, stems, inflorescences and seeds of each cultivar. Accordingly, we calculated: (i) root allocation, as the root/stem ratio; (ii) vegetative aboveground allocation, as the stem/reproductive component ratio (the reproductive component was obtained by summing inflorescence and seed dry weight); (iii) reproductive allocation, as the inflorescence/vegetative biomass ratio (vegetative biomass was obtained by summing root and stem dry weight); (iv) seed allocation, as the seeds/vegetative biomass ratio.

#### Leaf traits and plant resource-use strategy

Functional traits are indicators of plant performance responses to environmental factors [38]. Several axes of variation have been proposed, aimed at the description of the relationships between functional traits and environmental factors. A major axis is the ‘economics spectrum’, that reflects a trade-off between the investment of resources in further resource acquisition versus resource conservation [39].

To explore the resource-use strategies we measured plant traits descriptive of the conservative-acquisitive strategy [40]. “Conservative species” are slow growing species that invest resources in tissues density, while “acquisitive species” invest resources in fast growth and reproduction [41,42]. Conservative species are more adapted to cope with resource-poor environments, while acquisitive species, which are able to quickly use the resources when they are available, are more adapted to unpredictable and unstable conditions [43]. Specifically, we measured the following plant traits: (i) Leaf Area (LA, mm^2^), calculated as the one-sided area of a fresh leaf, (ii) Leaf Dry Matter Content (LDMC, %), as the ratio of leaf dry mass to fresh mass, and (iii) Specific Leaf Area (SLA, mm^2^ /mg), as the ratio of the leaf area to the leaf dry mass [41,43,44]. LA is linked to the plant capacity to intercept light and measures how much a plant invests in the photosynthesis [41,42]. Under stressful conditions, LA tends to decrease [44]. SLA increases at increasing growth rate, while lower values tend to correspond with investments in defense structures [41]. LDMC is related to the density of the leaf tissues; LDMC tends to be high in tough leaves and is inversely related to growth rate [41,44]. Plant traits were measures on 4 leaves of 5 adult individuals, without obvious symptoms of pathogen or herbivore attack, according to standard protocols [41,44].

### Metabolomic analysis

The metabolomic analysis aimed to identify the presence of bioactive compounds in the different parts of the plants and to highlight possible differences in the chemical profile of the four cultivars, with particular attention to the composition of seeds. The nutritional profile of seeds was outlined considering essential compounds (fatty acids, sugars, free amino acids) and potential bioactive compounds (phenolic amides, cannabisins, trigonelline). The chemical analysis was performed using the untargeted metabolomic approach, to identify a high number of interesting compounds. This approach, while not providing quantitative information, permits to compare the metabolic pattern of different groups of samples and to simultaneously verify the presence and relative abundance of many compounds, both known and unknown, without the need of standard substances.

Sampling activity has been conducted in July-August 2017. To perform metabolomics analysis, five randomly chosen individuals have been collected for each cultivar. To reduce variability and obtain a representative sample for each cultivar, the different parts of the plants (leaves, stems, roots, and flowers) have been unified. Seeds were collected at maturity during the month of September-October 2017.

Plants were stored at -20°C until analysis and then freeze-dried (Modulyo Edwards freeze-dryer), milled and homogenized by means of a ball mill (MM 400, Retsch, Verder Scientific, Haan, Germany). The procedure employed is based on the protocol of De Vos et al. [45]. The lyophilized and milled plant material was weighted (50± 0.5 mg), and the internal standard (IS, Leukine-Enkephalin, Sigma-Aldrich, Sant Louis, Missouri, USA) was added at a concentration of 30 ng/g. After homogenization, the samples were treated for 30 min in an ultrasonic bath with 1.5 mL of MeOH/H_2_O 75:25 (v/v) acidified with formic acid 0.1% and then centrifuged for 20 min at 14,000 rpm; the supernatant was collected, filtered with PTFE syringe filters (Ø 25 mm, 0.2 μm) and finally analyzed. To verify instrumental repeatability, 8 control mix samples were prepared as a pool of all the considered sample typologies, subjected to the same treatment and analyzed all throughout the instrumental sample series. Moreover, to verify the possible contamination during the sample treatment, we analyzed three blank samples, subjected to all the phases of sample processing.

#### Instrumental analysis

Analyses were performed by an UltiMate 3000 (Dionex) coupled to an ESI-LTQ Orbitrap XL (Thermo Fisher Scientific, Waltham, USA), following the method already described by Scalabrin et al. [46]. Briefly, a SB-Aq Narrow Bore RR 2.1×150 mm, 3.5 μm column (Agilent Technologies, Wilmington, USA) was used, eluted with H_2_O and ACN acidified with 0.01% of formic acid. The analyses were conducted in full-scan modality, in both positive and negative polarities, at a resolving power of 60,000 (mass range 90–1500 m/z). Data-dependent acquisitions were also performed to obtain a complete fragmentation pattern of the molecules. Measurements were carried out by means of the internal calibration method (reference mass) to automatically correct the mass calibration for each scan, obtaining a mass accuracy of 2 ppm. The intensity of the reference mass was monitored over samples to assure constant and reproducible ionization.

#### Chemicals and reagents

Ultra-grade methanol (MeOH) and ultra-grade acetonitrile (ACN) were purchased from Romil® LTD (Cambridge, UK), ultrapure water (18.2MΩ, 1ppb TOC) was produced using a Purelab Ultra System (Elga®, HighWycombe, UK), formic acid (≥98%) eluent additive for HPLC system was obtained from Fluka (Sigma Aldrich®, Buchs, Switzerland). Leucine-Enkephalin (Sigma-Aldrich, Sant Louis, Missouri, USA) solution was prepared by solid standard (purity ≥95%) and diluted in MeOH. Analytical standard of quercetin, apigenin and kaempferol were used to confirm metabolite identity (Sigma-Aldrich, Sant Louis, Missouri, USA).

#### Data elaboration

Mass spectra has been manually elaborated, to identify the detected compounds and particularly the more significant differences among cultivars. Metabolite identification was performed considering the monoisotopic mass, the most probable molecular formula, and the fragmentation pattern by means of XcaliburTM software (Thermo Scientific Inc.) by comparison with available online libraries (Metlin, HMDB, Dictionary of Natural Products, and LIPID MAPS Structure Database) and literature. The identification level was assigned according to Sumner et al. [47].

### Statistical analyses

Plant size, biomass allocation, plant functional traits (LA, SLA, LDMC), and the seed mass were compared among cultivars by PERMANOVA [48], followed by Post Hoc Tukey test. Moreover, to explore the trade-off between the number of seeds and seed mass, we compared the type of seed produced among the cultivars, as the ratio between the number of seeds produced and their weight.

Chemical data were normalized, and levels of primary and secondary metabolites were compared by the analysis of variance (one-way ANOVA test); differences were considered significant at a probability level of p < 0.05. The Metaboanalyst 5.0 software [49] was used to implement the statistical analysis and graphic presentation of metabolomic data performing a heatmap. Heatmap was built considering the sum of intensities of metabolites belonging to the different classes (cannflavins, flavonoids, glycosidic flavonoids and cannabinoids) and setting the following parameters: distance measure: Euclidean; clustering algorithm: Ward, standardization: autoscaling features, from log2 transformed and normalized data.

Analyses were performed with Past 3.0 [50] and Statistica 8.0 (StatSoft, Inc., 2007) softwares.

## Results

### Plant density, life cycle phases and plant size

Plant density ranged from 58 in CS to 122 seedlings/m^2^ in FEL (Table 2). Cultivars differed in plant size (Permanova test; 7.267; P = 0.0009), with CS showing the highest tallness (although not significantly different from FEL and FUT) and stem diameter.

**Table 2:** Plant density and size of each cultivar.

Cultivar	Plant density (n/m^2^)	Plant height (cm)				Stem diameter (mm)			
CS	58	113.6	±	23.5	a	5.0	±	1.3	a
FEL	122	112.7	±	21.6	a	3.8	±	0.7	b
FUT	108	98.4	±	12.8	ab	3.4	±	0.6	b
JUB	94	82.6	±	8.5	b	3.4	±	0.7	b

For each column, values with different letters are significantly different according to post-hoc Tukey test, at a significance level of p < 0.05.

**Fig 2:**
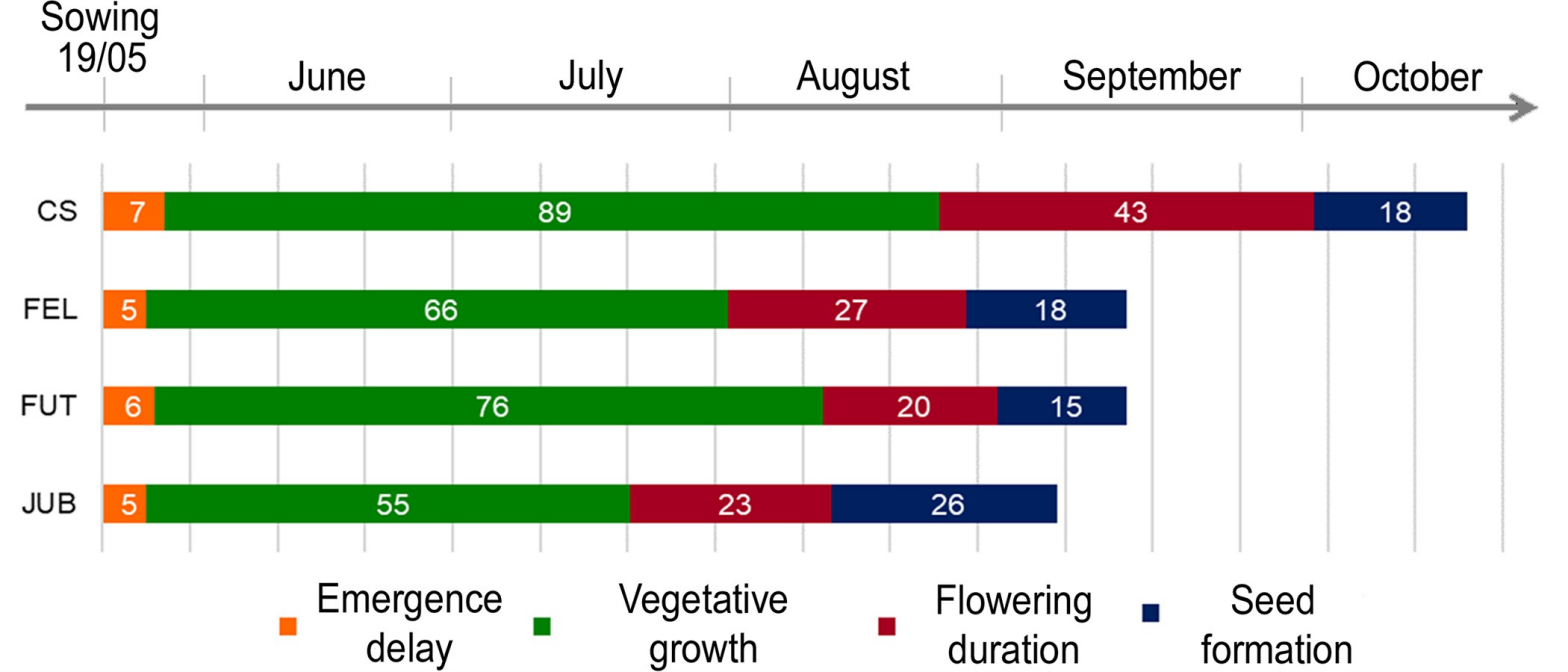
Phenological phases of each cultivar. Numbers are days elapsed from the beginning and the end of each phase.

Emergence delay was about 1 week for all cultivars (Fig 2). The four cultivars showed differences in phenological phases and growth. CS had the longest total duration of the life cycle (157 days) and a late transition from the vegetative to the reproductive phase, while JUB showed the shortest life cycle (109 days) and the earliest transition to flower formation. Seed maturation required between 15 and 26 days and occurred between September and October in all cultivars.

### Biomass allocation and leaf traits

**Fig 3:**
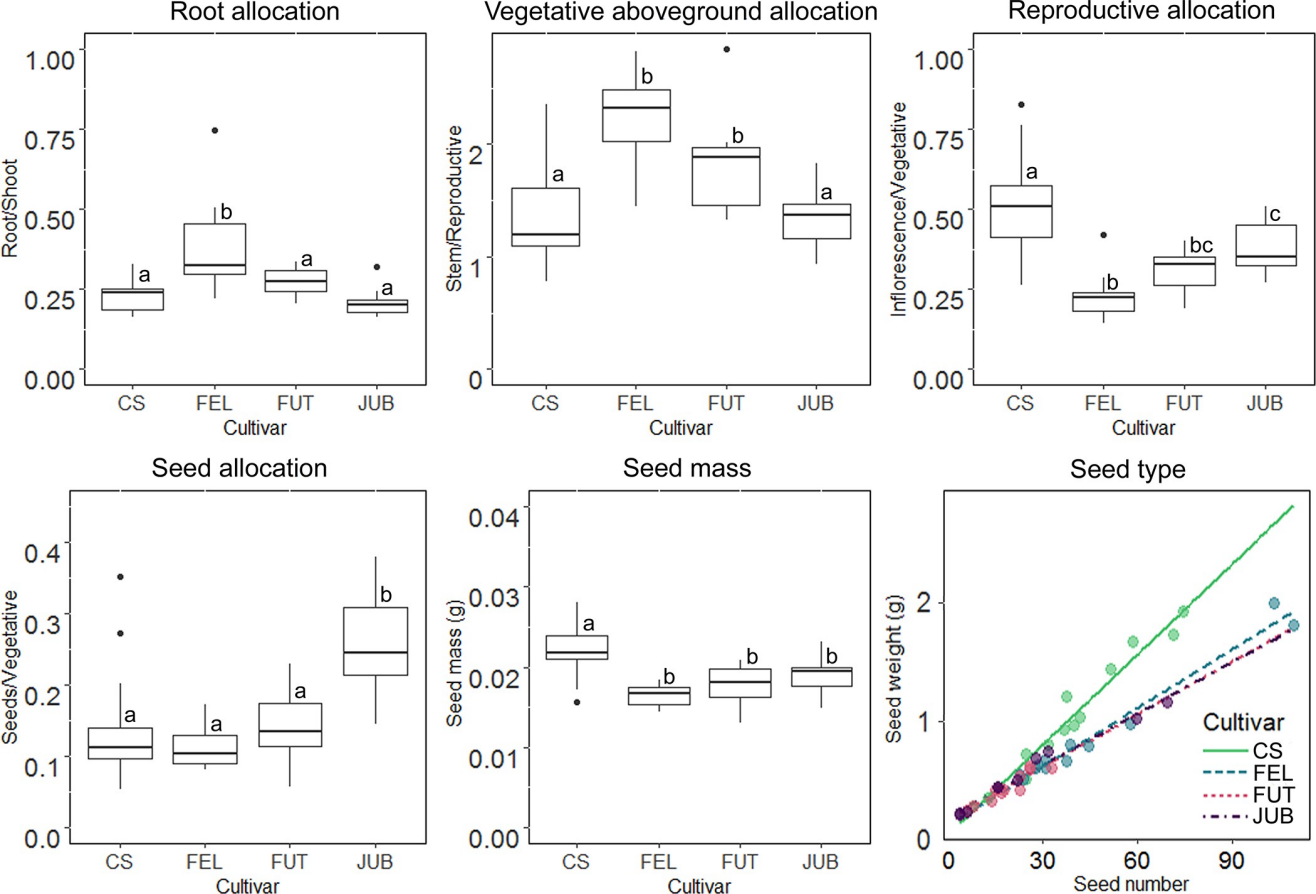
Biomass allocation. Biomass allocation in root, vegetative (stem and leaves), reproduction (inflorescence) and seeds, seed mass, and type of seed produced of the target cultivars. Boxes with different letters are significantly different according to post-hoc Tukey test, at a significance level of p < 0.05.

The cultivars differed in biomass allocation patterns (Permanova test; F = 13.71, P<0.001; Fig 3). FEL and FUT invested significantly more in vegetative aboveground structures (stem/reproductive component ratio) compared to CS and JUB. FUT also featured significantly higher levels of biomass allocated to the root. Across the cultivars, CS had the highest biomass allocation to reproductive (inflorescence) structures, while JUB significantly revealed as the cultivar with the highest allocation to seeds. Interestingly, seed mass was significantly higher in dioecious CS (Fig 3; S1 Table).

The four cultivars also differed in leaf traits and the resource-use strategy (Permanova test; F = 3.299, P = 0.04; Table 3). CS showed high values for both LA and SLA, coupled with low value for LDMC, revealing an acquisitive resource-use strategy. Conversely, FEL was characterized by the highest investment in leaf defense structures, that indicated a more conservative strategy. FUT and JUB had a less defined resource-use strategy, but with a tendence towards the acquisitive strategy (high SLA).

**Table 3:** Plant traits.

	LA (mm^2^)	SLA (mm^2^/mg)	LDMC (%)
** **CS** **	2212.06 ± 574.10 a	27.77 ± 1.71 a	0.28 ± 0.01 a
** **FEL** **	1589.46 ± 453.55 ab	22.46 ± 2.07 b	0.31 ± 0.02 b
** **FUT** **	1448.26 ± 666.14 ab	26.63 ± 2.15 a	0.29 ± 0.02 ab
** **JUB** **	1240.21 ± 287.06 b	25.81 ± 2.29 ab	0.28 ± 0.01 a

Means ± SD of LA, SLA, and LDMC among the four cultivars. For each column, values with different letters are significantly different according to post-hoc Tukey test, at a significance level of p < 0.05.

### Metabolomic analysis

The metabolomic analysis allowed to evidence differences in the chemical profile of the four cultivars. The complete list of identified compounds is in the Supporting information (S2 Table).

#### Flavonoids

**Fig 4:**
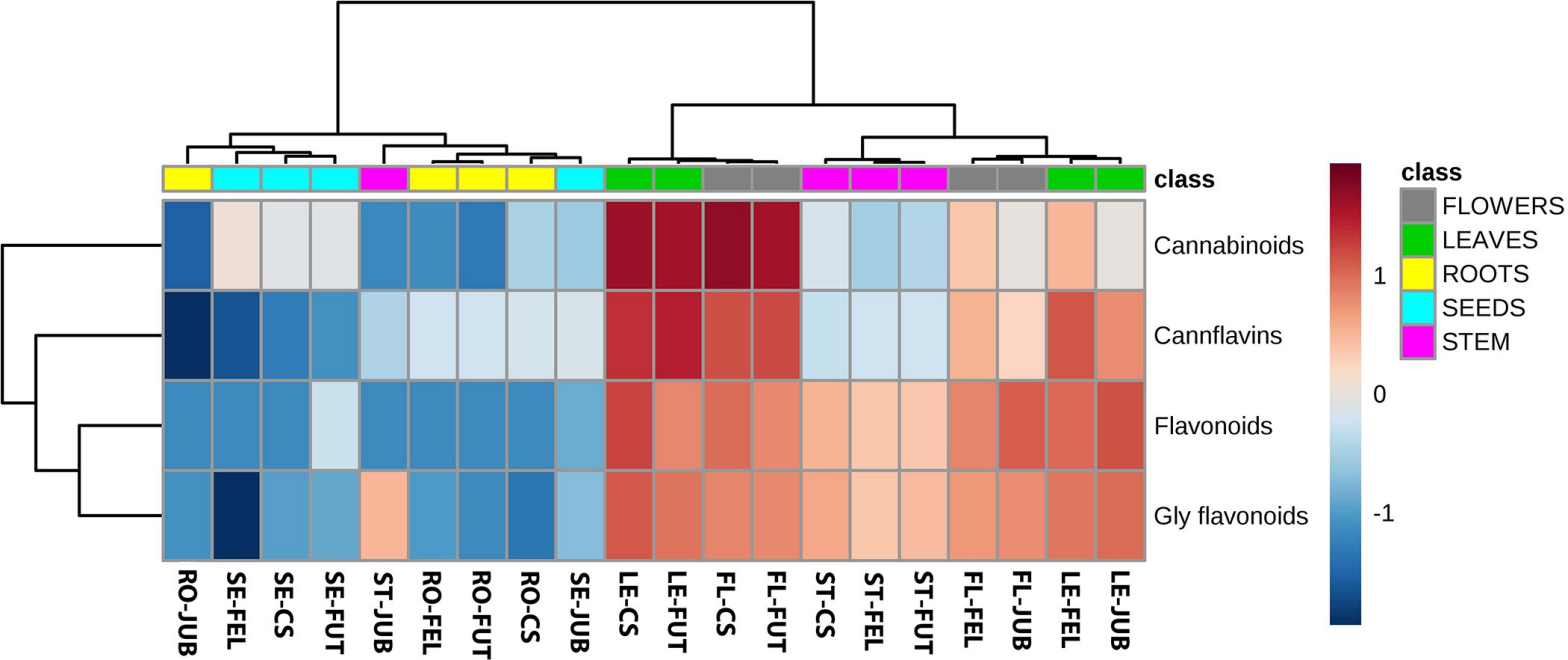
Metabolites heatmap. Heatmap of the selected metabolites analyzed in the different part of the plants (flowers, leaves, roots, seeds and stems). A color-coded matrix (from blue for the lower values to red for higher values) represents the intensities of the sum of metabolites detected for each class. Each sample is marked by two letters indicating the part of the plant (FL = flower, LE = leave, RO = root, SE = seed, ST = stem) and the variety acronym.

Three classes of flavonoids were found. Cannflavins (particularly Cannflavin A and B) showed to be present in all the four cultivars, with higher levels in FUT and CS (Fig 4). Interestingly, the compounds were distributed mainly in the leaves (49–54% of the total content of the plant), in the flowers (29–35%) and in the stems (14–19%). The other flavonoids were present in all the cultivars mainly in the glycosidic form; particularly apigenin, luteolin, kaempferol, quercetin glucuronide, apigenin glucuronide, apigenin rutinoside, apigenin glucoside, luteolin/kaempferol glucoside, luteolin/kaempferol rutinoside, luteolin/kaempferol glucuronide were detected. Both the glycosidic and the a-glycosidic flavonoids showed the highest levels in CS (Fig 4); again, the compounds were distributed among the leaves (42–58% and 48–54%), the flowers (31–45% and 28–35%) and the stems (0–13% and 14–17%) for the flavonoids and their glycosides respectively.

#### Cannabinoids

The qualitative analysis of cannabinoids permitted the identification of 11 masses belonging to this known class of compounds; the discrimination of isomers was not possible due to the similar fragmentation pattern of compounds. The 95–97% of total amount of cannabinoids in all cultivars were present in the acidic form: tetrahydrocannabinolic acid (THCA), cannabidiolic acid (CBDA), cannabinolic acid (CBNA), cannabichromene acid (CBCA), cannabigerolic acid (CBGA), cannabidivarinic acid (CBDVA), tetrahydrocannabivarinic acid (THCVA) were the major compounds detected. The highest level of cannabinoids was detected in CS, followed by FUT (Fig 4), FEL and JUB. The compounds were distributed mainly among flowers and leaves which contained the 34–57% and the 40–57% of cannabinoids respectively; CS presented higher levels of cannabinoids in the flowers than in the leaves, with 57% and 40% respectively, while FEL had higher levels in the leaves than in the flowers, with percentages of 57 and 34 respectively. In the other two cultivars the cannabinoids were distributed equally between flowers and leaves. In the seeds, small percentages of cannabinoids were detected, with the higher relative abundance for FEL (8%) and the lower for CS (2%).

#### Seed nutritional profile

**Fig 5:**
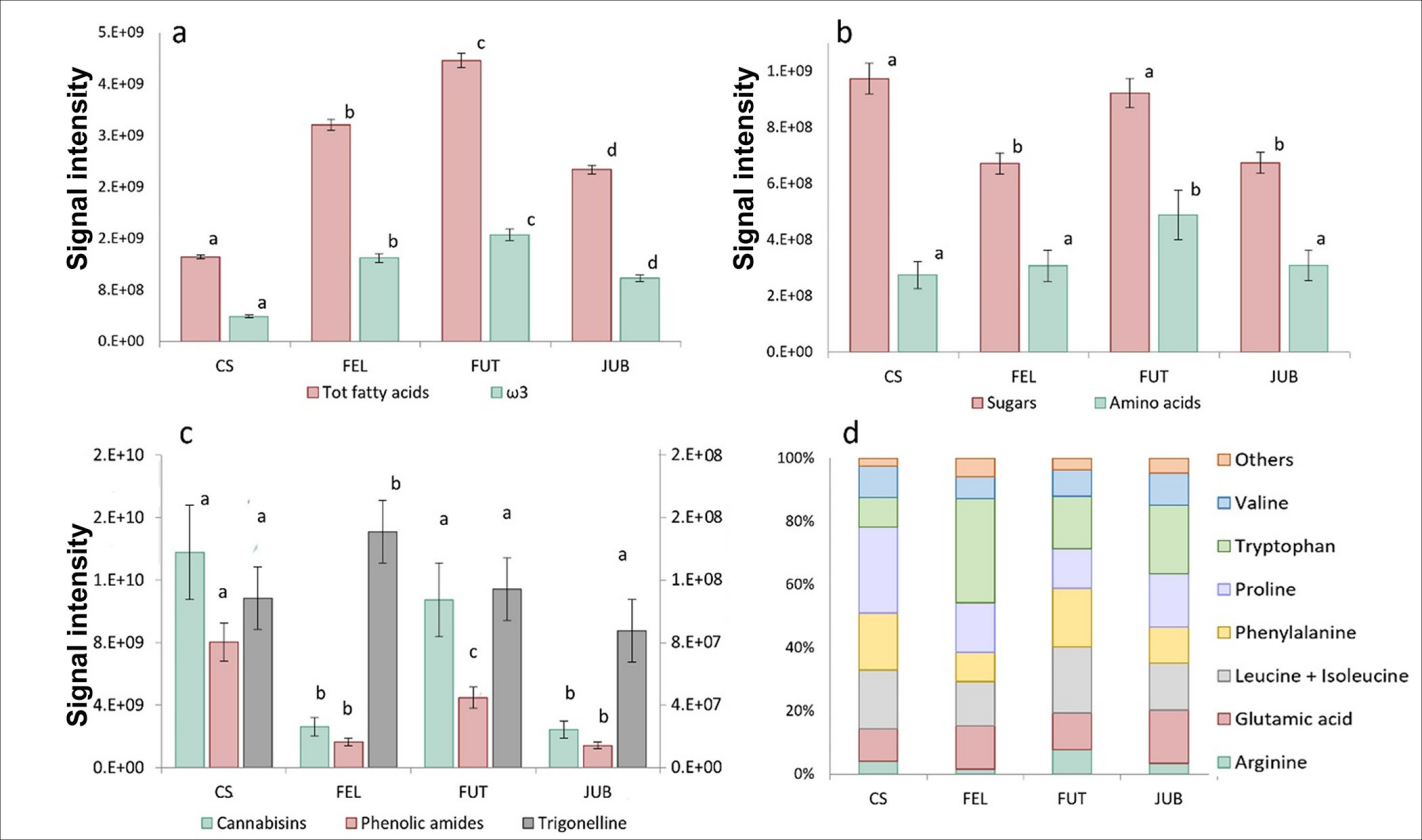
Fatty acids, ω3, amino acids, sugars, bioactive compounds. Levels of total fatty acids and ω3 (a), of amino acids and sugars (b), bioactive compound levels (cannabisins, phenolic amides and trigonelline) (c), single amino acid compounds percentages (d) in hemp seed samples. Error bars represent relative standard deviation among three samples. Statistically significant differences (T test, p value <0.05) are marked by different letters within the same data series.

The highest fatty acid level in our samples was observed in FUT. The lowest content was measured in CS, which presented 1/3 of the total fatty acid content of FUT (Fig 5a). However, the ratio between ω 6 and ω 3 was maintained in all cultivars, with values of 1.5–2.3.

The content of sugars (sucrose, glucose/inositol, xylose/arabinose/ribose, galatturonic acid) was also evaluated, with the highest levels in CS and FUT (Fig 5B). Sucrose represented the 99% of total sugars in all cultivars.

Total free amino acids presented the highest value in FUT while the content in the other cultivars resulted comparable (Fig 5B). The relative abundance of single compounds was similar in the cultivars (Fig 5D), with a higher percentage of tryptophan in FEL and a higher presence of proline in CS. Bioactive compounds were also identified in hemp seeds and particularly cannabisins, phenolic amides and trigonelline. Higher levels of cannabisins were found in FUT and CS while JUB and FEL showed lower levels (Fig 5C). Phenolic amides were present in higher levels in CS, followed by FUT, while JUB and FEL had lower intensities (Fig 5C). Trigonelline showed the higher levels in FEL while, among the others, there were no significant differences in its concentrations.

## Discussion

Land marginalization and abandonment arise from the interaction of several factors but anyhow lead to the loss of economic and ecological viability for local communities [51]. Developing innovative and sustainable production systems that allow for the development of a wide range of novel products and services is crucial to disclosing the potential of these sites. The multidisciplinary approach used in this study allowed identifying some differences between the four hemp cultivars selected for cultivation in marginal lands in the Alpine region of northern Italy, useful for a proper cultivar selection and product quality. Considering the urgent need for climate change mitigation and adaptation strategies, support the recultivation of marginal land by rebuilding sustainable high-quality and multi-functional agrosystems can contribute also to protect biodiversity and soil fertility, promoting ecosystem services and a better land-use management [52].

Despite hemp is often described as a crop suitable for a wide range of environmental conditions (e.g., [14,53]), our study evidenced significant differences among the four target cultivars regarding both their ecological and agronomic behavior and nutritional profile. Variation in life cycle and in the length of the different stages as well as in biomass allocation patterns and resource-use strategies can be caused by both environmental and genetic factors [54] suggesting that the interaction between genotype, environment and crop management is crucial in growing hemp [55]. In our study, both environmental variables and crop management were uniform across cultivars, suggesting a major role of genotype.

From the metabolomic point of view, our study confirmed hemp as an attractive non-food crop, that produces a wide variety of resources [56]. Building on this perspective, the advantages of incorporating hemp into industrial products are evident. For instance, the utilization of hempseed expellers to feed broiler chicken ameliorated the colour and odour of the meat [57], while the incorporation of hemp raw materials in bakery products enhanced several aspects, such as crust colour and crumb elasticity [58]. This approach aligns with the European Green Deal Farm to Fork Strategy and stimulates the introduction of low-input crops like hemp, potentially fostering the transition to a sustainable and competitive food system. Such a shift can capture new market opportunities, empower local communities, and contribute to valorising rural landscapes, aligning with the goal of recultivating abandoned lands for sustainable food production and economic profit [7,59].

Although hemp is traditionally grown for either fiber or seed/oil production, our study evidenced the presence of several compounds with potential pharmaceutical and nutraceutical values. Hemp seed oil is a valuable addition to human diet [16] and fatty acids are known as important constituents of hemp seeds; particularly α-linolenic and linoleic acids are the most abundant compounds of ω 6 and ω 3 classes respectively, known to be essential for humans [60]. These compounds must be obtained from diet, being not biosynthesized in the human body. Particularly important is the ratio between ω 3 and ω 6 fatty acids in food: a value ≈ 3 is considered to be optimal for nutrition, lowering cholesterol and high blood pressure for cardiovascular disease prevention [61].

In the seeds the presence of bioactive compounds as trigonelline, cannabisins and phenolic amides was highlighted. Trigonelline is an alkaloid, widely found in plants [62], to which are ascribed some bioactive properties, including the interactions with the prostaglandin EP2 receptors [63]. Cannabisins and phenolic amide are metabolites found in high quantity in *Cannabis* seeds, which showed to possess a number of bioactive effects as antioxidant, anti-inflammatory, cytotoxic activity against tumoral cells [64–66]. Moreover, other metabolites such as phenolics and flavonoids from industrial hemp showed to be promising for many nutraceutical and cosmetic applications: as adjuvant in the treatment of metabolic disorders [67], as antibacterial agents against *Escherichia coli* [68], as anti-oxidants and anti-inflammatory compounds [69,70]. Currently, the utilization of hemp metabolites for nutraceutical and pharmaceutical purpose in Italy is limited by the lack of clear legal frameworks for cultivated varieties, and unclear control procedures to ensure compliance with hemp cultivation regulations [10]. To advance more comprehensive research on hemp metabolites with potential pharmacological effects, there is a need for more explicit legislation in this domain.

The metabolomic analysis identified the presence of bioactive compounds in all the parts of the plants. Specifically, it evidenced three major classes of flavonoids: the cannflavins, which are methylated isoprenoid flavones typical of the genus *Cannabis*, with antioxidant and anti-inflammatory activities [71,72]; three flavons and a flavonols known to be present in *Cannabis* (namely Apigenin, Luteolin, Kaempferol and Quercetin) and their glycosidic forms (glucosides, rutinosides, glucuronides). As expected [73], other flavonoids were present in all the cultivars mainly in the glycosidic form. Cannabinoids were also present in all cultivars and in all parts of the plants, mainly in flowers and leaves. They are present in the acidic form, confirming previous studies [62,74,75]; acidic cannabinoids can be decarboxylated to their neutral homologues which are the main bioactive and psychoactive forms [74], both within the plant and, to a much larger extent, upon heating after harvesting [73].

The most interesting result of this study regards the importance of all parts of the plant, and particularly leaves, followed by the inflorescences and stems. This outcome, coupled with the different biological and ecological behaviour of the target cultivars can help select the most suitable cultivar according to the end-use.

Among the selected cultivars, JUB was smaller and allocated the highest proportion of biomass to seed production in comparison to the other cultivars. This could be due to JUB early flowering period which hinders vegetative growth, redirecting resources to reproductive structures. Indeed, early flowering cultivars are generally better suited for grain production [30]. Being short, JUB is expected to be buffered from lodging better than the other cultivars, and could be cultivated also in sites with intense rain and wind [53,76] like hilly and mountain regions. However, from a qualitative point of view, the nutritional profile of JUB seeds (if compared to CS and FUT) showed the lowest content of bioactive compounds (cannabisins and phenolic amides), while the amount of essential compounds (amino acids, sugars and fatty acids) was as high as those of FEL and CS. For what concern the other parts of the plant, JUB had the lowest quantity of cannabinoids and cannflavins in flowers and leaves compared to other cultivars, but the content of glycosidic flavonoids was similar to those of FEL and FUT. For these reasons, JUB confirms to be more suited to the production of seeds than of inflorescences and leaves.

CS cultivar was late flowering, had the lowest plant density, and produced tall plants. This is in agreement with previous studies which reported that late flowering and low plant density maximize stem size [33,34]. Low density and plant height suggest that CS could be more sensitive to weed invasion and lodging compared to the other cultivars [4,53,76], while leaf traits, which indicated a predisposition toward a fast growth and acquisitive strategy, suggest that this cultivar could be suitable for cultivation in unstable environments [40]. CS also stood out for the production of seeds of high mass. From a qualitative standpoint, the seeds of CS had the highest content of cannabisins, which have shown to possess antioxidant and acetylcholinesterase inhibitory activities [77], and phenolic amides, which are known to possess anti-inflammatory, antioxidant and chemopreventive activities [62,78]. The highest biomass allocation in CS cultivar was in the inflorescence. This result could be also attributed to the lower density of plants in CS plot, considering that plant shape and the distribution of dry matter to organs and tissues are strongly affected by plant density and, to a lesser extent, by cultivar [79]. Qualitative analysis on CS inflorescences showed the highest content of cannabinoids, cannaflavins and glycosidic flavonoids, which are distributed mainly in the leaves and the flowers (40% and 57% for cannabinoids respectively, 60% and 37% for cannaflavins, and 54% and 28% for glycosidic flavonoids); these compounds have shown to possess a wide range of biological activities, including antioxidant, antimicrobial and neuro-protective [62,69]. A previous study [75] indicated altitude as a favorable climatic condition for the accumulation of cannflavins in *Cannabis sativa* flowers. The presence of flavonoids is known to be highly related to UV light and temperatures since they are involved in plant protection mechanisms as radical scavengers. Therefore, CS could be suitable for the production of inflorescence, to be used in nutraceutical and herbalist applications; however, it must be considered that this cultivar, being dioecious, has a lower productivity of inflorescence than monoecius cultivars (e.g., FUT).

A very important result, indeed, is represented by the presence of bioactive principle in CS leaves, which currently represent a byproduct of hemp production. The possibility of extract and employ these compounds as supplement could provide high added value to hemp production, extremely increasing economic sustainability of this culture. Furthermore, the production of inflorescence and the extraction of bioactive principles from leaves could be effectively combined, because harvesting is carried out at full flowering, when the number of leaves reaches its maximum. These kinds of applications could stimulate hemp cultivation in marginal areas, since growers could maximize the revenues that can be obtained from a medium or small-sized plot.

FUT showed good values of bioactive compounds (cannabinoids, cannflavins and flavonoids) in the plant in comparison to the other cultivars. Particularly, FUT showed the highest content of cannflavins and a good content of glycosidic flavonoids, proving to possess a high potential for bioactive compounds extraction. The biochemical profile of FUT seeds showed the highest levels of both nutritional compounds (fatty acids, total free amino acids, sugars) and bioactive substances (cannabisins and phenolic amides) demonstrating a high potential also for seed production. Considering the percentage of biomass production, FUT showed the most balanced distribution among stem, flowers, and seeds. Therefore, FUT seems to be suitable for multipurpose hemp cultivation, that goes from the exploitation of the stem for green building applications up to the extraction of high added value products. This cultivar could represent a good compromise in terms of biomass productivity, nutritional and bioactive content and be particularly advantageous in marginal lands where the net yield is generally lower than in other areas, permitting the use of different part of the plant and providing an important added values from the extraction of bioactive principles.

FEL combined high plant density with tall plant height, and biomass allocation in roots and vegetative parts of the plant. The high biomass allocation to roots could contribute to counteract plant lodging through an efficient anchorage in the soil, while the high plant density could contribute to counteract weed invasion [4]. Leaf traits indicated a conservative resource-use strategy, which reflects an investment in structural components rather than in leaf growth. Despite a relatively early flowering (at the beginning of August), FEL had a low biomass allocation to seeds, and a low seed mass. This could be a consequence of the high plant density, which may had favored stem length to the detriment of branch and flowers development [15]. All these features suggest the suitability of FEL for fiber production, rather than for seed use. However, the investment in structural components and the low seed mass could also suggest that FEL may have suffered high temperature stress, which is known to determine low productivity [80] and a decrease of photosynthesis activity [81]. Indeed, in the study area, the daily temperature can exceed 28°C during July and August [82]. FEL is known to be particularly adapted to Atlantic climate, being often cultivated in Northern Europe [83,84], resulting less suitable for Mediterranean region [81,85]. Therefore, FEL seems to be not as suitable for the cultivation in northern Italy Alpine marginal areas as the other cultivars.

## Conclusions

The multi-disciplinary approach we adopted was effective in distinguish the characteristics of each hemp cultivar, valorizing the peculiarity of each one, suggesting the possibility to extend this approach to other marginal areas. The results of the study highlight the potentiality of hemp culture in northern Italy Alpine marginal areas and could serve as a stimulus for the reintroduction of this crop in this area and in other similar environments.

Despite our results are relative to only one year of cultivation and need to be confirmed by further studies, our experience revealed that three out of four cultivars demonstrated better suitability for cultivation in the study area. Therefore, our results suggest that to effectively recultivate marginal lands abandonment through hemp introduction, the cultivar selection must be carefully considered based on the local environment. Additionally, our study confirms that hemp cultivation offers the opportunity to develop a wide range of new products and services, serving as an attractive crop for the production of bioactive resources with pharmaceutical and nutraceutical potential.

However, to optimize the use of hemp in marginal areas, there is a need to invest in more efficient methods for harvesting and processing this crop, as well as to establish clearer legislation regarding the legality of cultivated varieties. Implementing such measures can maximize the positive impact on socioeconomic aspects and contribute to the effective development of marginal lands.

## Supporting information

S1 Table Cultivar morphology. Means ± SD of the morphological parameters measured for each cultivar.
(DOCX)

S2 Table Metabolites. Name, acronym, molecular formula, precursor ions in positive and negative polaritites, detection polarity, fragments and identification level of the analyzed metabolites.
(DOCX)
