# Supplementary material for: Hemp cultivation opportunities for marginal lands development
Source: PLoS One. 2024 Mar 21;19(3):e0299981. doi: 10.1371/journal.pone.0299981 (PMC10956763; doi:10.1371/journal.pone.0299981)
Supplement: S1 Table — Means ± SD of the morphological parameters measured for each cultivar. (DOCX) [file pone.0299981.s001.docx]

**S1** **Table. Cultivar morphology.** Means ± SD of the morphological parameters measured for each cultivar. Different letters indicate significant differences to post hoc Tukey test, at a significance level of p < 0.05.

To describe the morphology of each cultivar we measured on 10 individuals (i) the length of the 4^th^ internode, to determine the length of the middle of the stem; (ii) the maximum number of leaves, achieved before flowering; (iii) the seed size (length, width and thickness), measured on 5 seeds from 5 plants per cultivar, by using a binocular microscope; (iv) the seed mass; (v) the seed shape; (vi) the number of seeds produced by each plant.

The seed shape has been calculated as the variance among length, width and thickness, after each of these values had been standardized, by dividing each value by the largest value (Thompson et al., 1993); the shape values vary between 0 and 0.3, which indicates spherical or cylindrical/flattened shapes, respectively. The “Seed number on seed weight ratio” is the number of seeds divided by their weight in grams.

| **Cultivar** | **Internode lenght (cm)** | **Number of leaves** | **Seed length (mm)** | **Seed width (mm)** | **Seed thickness (mm)** | **Seed mass (mg)** | **Seed shape** | **Seed number** | **Range of the number of seeds (min-max)** | **Seed number on seed weight ratio** |
| --- | --- | --- | --- | --- | --- | --- | --- | --- | --- | --- |
| CS | 10.2 ± 1.6b | 15.1 ± 1.3ab | 5.1 ± 0.3a | 4.1 ± 0.3a | 3.4 ± 0.2a | 22.1 ± 3.3a | 0.029 ± 0.010b | 40.8 ± 19.7ab | 9-79 | 46.3 ± 7.5b |
| FEL | 15.4 ± 3.9a | 15.6 ± 2.2ab | 4.8 ± 0.3b | 3.5 ± 0.2c | 2.7 ± 0.2c | 16.5 ± 1.3b | 0.044 ± 0.014a | 47.7 ± 28.6a | 24-111 | 61.1 ± 5.0a |
| FUT | 13.1 ± 2.1ab | 13.8 ± 2.2b | 4.8 ± 0.3b | 3.6 ± 0.2b | 2.9 ± 0.2b | 17.7 ± 2.3b | 0.035 ± 0.008b | 19.8 ± 7.5b | 7-34 | 57.6 ± 8.4a |
| JUB | 11.5 ± 2.6b | 16.0 ± 3.2a | 4.6 ± 1.7b | 3.3 ± 1.2c | 2.7 ± 1.0c | 18.9 ± 2.4b | 0.048 ± 0.012a | 26.8 ± 21.7ab | 5-71 | 53.9 ± 7.5ab |

**References**

Thompson, K., Band, S., Hodgson, J., 1993. Seed size and shape predict persistence in soil. Funct. Ecol. 7, 236–241. https://doi.org/10.1017/CBO9781107415324.004
