# Supplementary material for: Hemp cultivation opportunities for marginal lands development
Source: PLoS One. 2024 Mar 21;19(3):e0299981. doi: 10.1371/journal.pone.0299981 (PMC10956763; doi:10.1371/journal.pone.0299981)
Supplement: S2 Table — Name, acronym, molecular formula, precursor ions in positive and negative polaritites, detection polarity, fragments and identification level of the analyzed metabolites. (DOCX) [file pone.0299981.s002.docx]

**S2 Table. Metabolites.** Name, acronym, molecular formula, precursor ions in positive and negative polaritites, detection polarity, fragments and identification level of the analyzed metabolites.

| Name | Acronym | Molecular formula | M -H | M + H | Rt | Detection Polarity | Fragments | ID Level |
| --- | --- | --- | --- | --- | --- | --- | --- | --- |
| Cannabinoids | | | | | | | | |
| Tetrahydrocannabinol acid + cannabidiolic acid + Cannabinolic acid + Cannabichromenic acid + Tetrahydrocannabivarin | THCA+CBDA+CBNA+CBCA+THCV | C_22_H_30_O_4_ | 357.2071 | 359.2216 | 34.28 | neg | 313.21674, 267.21173, 204.11559, 179.10780, 173.09737, 158.07401 | 3 |
|  |  |  |  |  | 36.83 | neg | 339.19681, 313.21735, 191.10760, 179.10751, 173.09702, 245.15443, 201.16467 | 3 |
|  |  |  |  |  | 37.29 | neg | 339.19681, 313.21735, 229.12280, 179.10751 | 3 |
| Cannabigerolic acid | CBGA | C_22_H_32_O_4_ | 359.2227 | 361.2373 | 34.51 | neg | 341.21198, 315.23291, 191.10773, 179.10770, 136.05286 | 2 |
| Cannabidivarinic acid + Tetrahydrocannabivarinic acid | CBDVA+THCVA | C_20_H_26_O_4_ | 329.1758 | 331.1903 | 31.95 | neg | 311.16507, 285.18582, 256.11053, 241.08719 | 3 |
|  |  |  |  |  | 34.64 | neg | 311.16513, 285.18582, 245.15454, 217.12370, 173.09741, 151.07671, 163.07664 | 3 |
| Cannabidiol + Cannabichromene + Cannabicyclol + Tetrahydrocannabinol | CBD+CBC+CBL+THC | C_21_H_30_O_2_ | 313.2173 | 315.2318 | 34.28 | neg | 267.21173, 245.15462, 204.11559, 179.10780, 173.09737, 158.07401 | 3 |
|  |  |  | 313.2173 | 315.2318 | 36.83 | neg | 245.15460, 191.10760, 179.10767, 173.09715, 230.13130, 201.16487, 107.05036 | 3 |
|  |  |  | 313.2173 | 315.2318 | 37.25 | neg | 295.20581, 267.21112, 229.12300, 191.10747, 179.10750, 173.09703 | 3 |
| Cannabinol + cannabinodiol | CBN+CBND | C_21_H_26_O_2_ | 309.1860 | 311.2005 | 32.03 | neg | 279.13879, 267.17526, 211.11282, 171.08162, 159.08138, 137.09717 | 3 |
|  |  |  |  |  | 36.23 | neg | 279.13879, 267.17526, 211.11282, 171.08162, 159.08138, 137.09718 | 3 |
|  | CBG | C_21_H_32_O_2_ | 315.2329 | 317.2475 | 34.59 | neg | 297.22180, 191.10773, 179.10770, 193.12338, 205.12337, 136.05286 | 2 |
| Cannabielsoin | CBE | C_21_H_30_O_3_ | 329.2122 | 331.2267 | 27.9 | neg | 329.2125, 311.2020, 271.1706, 205.1233, 179.1066 | 2 |
| Cannabitriol | CBT | C_21_H_30_O_4_ | 345.2071 | 347.2216 | 27.59 | neg | 271.1695, 207.1015 | 2 |
| Cannabivarin | CBV | C_19_H_22_O_2_ | 281.1547 | 283.1692 | 35 | neg | 195.2649, 227.2635 | 2 |
| Cannabidivarin + Cannabichromevarin | CBDV+CBCV | C_19_H_26_O_2_ | 285.1860 | 287.2005 | 31.9 | neg | 217.1230, 285.1857, 163.0756, 151.0748 | 3 |
|  |  |  |  |  | 34.66 | neg | 217.1233, 285.1859, 163.0752 | 3 |
| Cannabigerovarin | CBGV | C_19_H_28_O_2_ | 287.2016 | 289.2162 | 35.02 | neg | 217.1234, 163.0768,151.0763 | 2 |
| Cannabigerolmonoethylether | CBGM | C_22_H_34_O_2_ | 329.2486 | 331.2631 | 23.74 | neg | 329.2488, 259.1702, 205.1231, 193.1236 | 2 |
| Cannflavins | | | | | | | | |
| Cannflavin B |  | C_21_H_20_O_6_ | 367.1187 | 369.1332 | 30.79 | pos | 313.07080, 298.04736, 165.01852 | 2 |
| Cannflavin A |  | C_26_H_28_O_6_ | 435.1813 | 437.1958 | 34.36 | pos | 313.07104, 298.04767, 165.01851 | 2 |
| Flavonoids | | | | | | | | |
| Apigenin |  | C_15_H_10_O_5_ | 269.0455 | 271.0600 | 24.81 | neg | 225.05545, 201.05518, 183.04472, 149.02400, 210.56505, 197.06044, 181.06561, | 1 |
| Quercetin |  | C_15_H_10_O_7_ | 301.0353 | 303.0499 | 22.79 | neg | 178.9986, 151.0037 | 1 |
| Luteolin |  | C_15_H_10_O_6_ | 285.0404 | 287.0550 | 18.46 | neg | 257.04501, 241.05017, 217.05031, 199.03958, 175.03961, 151.00345 | 2 |
| Kaempferol isomer |  | C_15_H_10_O_6_ | 285.0404 | 287.0550 | 22.47 | neg | 271.0627, 257.04501, 241.05017, 217.05031, 199.03958, 175.03961, 151.00345 | 2 |
| Luteolin/Kampferol glucoside |  | C_21_H_20_O_11_ | 447.0933 | 449.10738 | 17.38 | neg | 285.0403 | 3 |
| Luteolin/Kampferol glucoside |  | C_21_H_20_O_12_ | 447.0933 | 449.10738 | 18.58 | neg | 285.0402 | 3 |
| Luteolin/Kampferol rutinoside |  | C_27_H_30_O_16_ | 609.1461 | 611.16066 | 16.84 | neg | 489.10184, 327.04968, 357.06003, 429.08081, 299.05490, 285.03931 | 3 |
| Luteolin/Kampferol glucuronide |  | C_21_H_18_O_12_ | 461.0725 | 463.0871 | 18.45 | neg | 285.03995, 297.03925, 339.04959 | 3 |
| Apigenin glucoside |  | C_21_H_20_O_10_ | 431.0984 | 433.11292 | 18.07 | neg | 283.06006, 311.05502, 341.06519, 323.05460, 269.04434 | 3 |
| Apigenin rutinoside |  | C_27_H_30_O_14_ | 577.1563 | 579.17083 | 17.76 | neg | 327.9679, 311.05472, 269.04479, 136.0165 | 3 |
| Apigenin glucuronide |  | C_21_H_18_O_11_ | 445.0776 | 447.09218 | 19.58 | neg | 269.04477, 325.07068, 175.02455, 297.07565 | 3 |
| Phenolic amides | | | | | | | | |
| Caffeoyltyramine |  | C_17_H_17_NO_4_ | 298.1085 | 300.123 | 20.01 | neg | 284.03195, 135.04501, 161.02409, 178.05058 | 3 |
| Feruloyltyramine |  | C_18_H_19_NO_4_ | 312.1241 | 314.1387 | 21.94 | neg | 297.09949, 178.05048, 135.04491, 270.11237, 148.05252 | 3 |
| Cumaroyltyramine |  | C_17_H_17_NO_3_ | 282.1136 | 284.1281 | 21.65 | neg | 162.05559, 145.02916, 119.05016, 134.06090 | 3 |
| Cannabisins | | | | | | | | |
| Cannabisin A |  | C_34_H_30_N_2_O_8_ | 593.1929 | 595.2075 | 20.84 | neg | 456.10730, 430.12823, 291.02890, 319.02350, 428.11194, 349.05734,293.04425, | 2 |
| Cannabisin B |  | C_34_H_32_N_2_O_8_ | 595.2086 | 597.2231 | 20.72 | neg | 456.14380, 456.10770, 485.17032, 322.10715, 269.08093, 319.02350, 349.05716, 263.03403 | 2 |
| Cannabisin C |  | C_35_H_34_N_2_O_8_ | 609.2242 | 611.2388 | 21.73 | neg | 446.15863, 499.18491, 283.09628, 336.12222, 269.08090, 322.10669, | 2 |
| Cannabisin D |  | C_3_6H_36_N_2_O_8_ | 623.2399 | 625.2544 | 22.92 | neg | 460.17432, 446.15875, 336.12238, 283.09625, | 2 |
| Cannabisin E |  | C_36_H_38_N_2_O_9_ | 641.2505 | 643.265 | 23.62 | neg | 489.20081, 328.11752, 591.21027, 551.25165, 460.17352, 432.17929, 369.14374, 312.12286, | 2 |
| Cannabisin F |  | C_36_H_36_N_2_O_8_ | 623.2399 | 625.2544 | 25.86 | neg | 460.17459, 297.11200, 486.15350, 283.09647, 445.15082,282.08884 | 2 |
| Lyciumamide D |  | C_34_H_34_N_2_O_8_ | 597.2242 | 599.2388 | 20.04 | neg | 433.14655, 486.17307,298.10724, 269.08090, 322.10693, 323.11035, 349.08954 | 2 |
| Trigonelline |  | C_7_H_7_NO_2_ | 136.0404 | 138,0549550 | 1.67 | pos |  |  |
| Sugars | | | | | | | | |
| Saccharose |  | C_12_H_22_O_11_ | 341.1089 | 343.1235 | 1.68 | neg | 179.05603, 161.04546, 143.03493, 119.03493, 89.02460 | 3 |
| Glucose, inositol |  | C_6_H_12_O_6_ | 179.0561 | 181.0707 | 1.73 | neg | 161.04564, 71.01431, 59.01431 | 3 |
| Xilose, arabinose, ribose |  | C_5_H_10_O_5_ | 149.0455 | 151.0601 | 1.69 | neg | 131.03511, 89.02476, 73.03007, 59.01449 | 3 |
| Galacturonic acid |  | C_6_H_10_O_7_ | 193.0354 | 195.0499 | 1.69 | neg | 113.0245, 85.0299 | 3 |
| Amino acids | | | | | | | | |
| Arginine |  | C_6_h_14_N_4_O_2_ | 173,1043990 | 175,1189522 | 1.54 | pos | 70.0651, 115.9642 | 2 |
| Glutamate |  | C_5_H_9_NO_4_ | 146,0458810 | 148,0604343 | 1.59 | pos | 84.0442 | 2 |
| Leucine + IsoLeucine |  | C_6_H_13_NO_2_ | 130,0873520 | 132,1019052 | 1.7 | pos | 86.09627 | 3 |
| Phenylalanine |  | C_9_H_11_NO_2_ | 164,0717019 | 166,0862552 | 1.7 | pos | 120.08006, 147.04329 | 2 |
| Proline |  | C_5_H_9_NO_2_ | 114,0560518 | 116,0706051 | 1.67 | pos | 70.06516 | 2 |
| Triptophan |  | C_11_H_12_N_2_O_2_ | 203,0826009 | 205,0971542 | 1.71 | pos | 188.06961, 146.05957 | 2 |
| Valine |  | C_5_H_11_NO_2_ | 116,0717019 | 118,0862552 | 1.67 | pos | 72.0809 | 2 |
| Tyrosine |  | C_9_H_11_NO_3_ | 180,0666165 | 182,0811698 | 1.65 | pos | 136.07572 | 2 |
| Serine |  | C_3_H_7_NO_3_ | 104,0353164 | 106,0498696 | 1.57 | pos | 60.0445 | 2 |
| Threonine |  | C_4_H_9_NO_3_ | 118,0509664 | 120,0655197 | 1.55 | pos | 74.06005 | 2 |
| Methionine |  | C_5_H_11_NO_2_S | 148,0437726 | 150,0583259 | 1.73 | pos | 56.04975 | 2 |
| Histidine |  | C_6_H_9_N_3_O_2_ | 154,0621998 | 156,0767531 | 1.59 | pos | 110.07132 | 2 |
| Aspartate |  | C_4_H_7_NO_4_ | 132,0302310 | 134,0447842 | 1.56 | pos | 74.02378 | 2 |
| Alanine |  | C_3_H_7_NO_2_ | 88,0404018 | 90,0549550 | 1.56 | pos |  | 2 |
| Lysine |  | C_6_H_14_N_2_O_2_ | 145,0982510 | 147,1128042 | 1.59 | pos | 84.08076 | 2 |
| Fatty acids | | | | | | | | |
| Palmitoleic acid |  | C_16_H_30_O_2_ | 253,2173035 | 255,2318567 | 35.88 | pos | 235.20699, 209.15497 | 2 |
| Palmitic acid |  | C_16_H_32_O_2_ | 255,23295 | 257.24751 | 37.33 | pos | 237.22221 | 2 |
| Oleic acid |  | C_18_H_34_O_2_ | 281,24860 | 283.26316 | 38.06 | pos | 262.22589 | 2 |
| Omega-3 | | | | | | | | |
| Alfa-linolenic acid | ALA | C_18_H_30_O_2_ | 277,21730 | 279.23186 | 35.4 | neg | 259.20612, 233.22702 | 2 |
| Stearidonic acid | SDA | C_18_H_28_O_2_ | 275,2016534 | 277,2162067 | 34.46 | neg | 231.21205, 177.16498 | 2 |
| Timnodonic acid | EPA | C_20_H_30_O_2_ | 301,2173035 | 303,2318567 | 35.45 | neg | 285.2214 | 2 |
| Eneicosapentaenoic acid | HPA | C_21_H_32_O_2_ | 315,2329535 | 317,2475068 | 34.92 | neg | 191.10721, 179.10725, 136.05270 | 2 |
| Tetracosapentaenoic acid |  | C_24_H_38_O_2_ | 357,2799037 | 359,2944570 | 34.32 | neg | 339.19644, 313.21713, 289.14444, 295.20660, 271.13406 | 2 |
| Omega-6 | | | | | | | | |
| Linoleic acid |  | C_18_H_32_O_2_ | 279,23295 | 281.2475 | 36.49 | neg | 261.22241 | 2 |
| Eicosadienoic acid |  | C_20_H_36_O_2_ | 307,2642537 | 309,2788069 | 38.5 | pos | 291.2685, 273.2585 | 2 |
| Docosadienoic acid |  | C_22_H_40_O_2_ | 311,2955538 | 313,3101070 | 41.38 | neg |  | 2 |
|  |  |  |  |  |  |  |  |  |
